# Impact of a visual indicator on the noise level in an emergency medical dispatch centre - a pilot study

**DOI:** 10.1186/s12873-021-00415-5

**Published:** 2021-02-23

**Authors:** Justin Outrey, Jean-Baptiste Pretalli, Sophie Pujol, Alice Brembilla, Thibaut Desmettre, Christophe Lambert, Jean-Marc Labourey, Frédéric Mauny, Abdo Khoury

**Affiliations:** 1 Department of Emergency Medicine and Critical Care, Besançon University Hospital, Boulevard Fleming, 25030 Besançon cedex, France; 2 INSERM CIC 1431, Besançon University Hospital, Besançon, France; 3 UMR Chrono-environnement 6249, CNRS/University of Bourgogne Franche-Comté, 25000 Besançon, France

**Keywords:** Medical call Centre, Noise exposure, Noise

## Abstract

**Background:**

Noise levels are monitored in call centres. A maximum of 52 to 55 dB(A) is recommended in order to prevent adverse events. We aimed at assessing the noise level and the impact of a visual noise indicator on the ambient noise level in a French Regional Emergency Medical Dispatch Centre (EMDC).

**Methods:**

We conducted an observational study in the EMDC of the SAMU25 (University Hospital of Besancon). We measured the noise level using a SoundEarII® noise indicator (Dräger Medical SAS, France). The measurement took place in two phases on three consecutive days from 00:00 to 11:59 PM. At baseline, phase 1, the device recorded the average ambient noise for each minute without visual indication. Secondly, phase 2 included a sensor mounted with a light that would turn on green if noise was below 65 dB(A), orange if noise ever exceeded 65 and red if it exceeded 75 dB(A).

**Results:**

In the presence of the visual noise indicator, the L_Aeq_ was significantly lower than in the absence of visual noise indicator (a mean difference of − 4.19 dB; *P* < 10^–3)^. It was higher than 55 dB(A) in 84.9 and 43.9% of the time in phases 1 and 2, respectively.

**Conclusions:**

The noise levels were frequently higher than the standards, and sometimes close to recommended limits, requiring preventive measures. The noise indicator had a positive effect on the ambient noise level. This work will allow the implementation of effective prevention solutions and, based on future assessments, could improve operators’ well-being and better care for patient.

## Background

The activity of emergency medical dispatch centres (EMDC) in France is constantly increasing due to the ageing population, the decrease in the number of healthcare professionals, lack of resources, but also the environmental and industrial changes that lead to an increase in major disasters and health crises. Moreover, people constantly seek for more performant acute and emergency care. In response, the number of emergency medical dispatchers (EMD) was increased [1]. EMDs are working with emergency physicians in a limited space and in an increasingly noisier environment.

The noise effects have already been studied in call centres providing customer services. Noise is a primary hazard threatening human health [2]. It can affect physical and mental health [3–5]. Noise can cause hearing loss, stress, discomfort and musculoskeletal disorders [6–14]. A noisy environment is incompatible with undisturbed intellectual work [15]. Moreover, 16% of adult hearing loss worldwide is related to noise exposure [16]. Call centres operators may also be subject to accidental high-intensity noises associated with the use of headsets (i.e. acoustic shocks) [17], which could induce a startle effect and a temporary hearing loss [5, 18]. Standards and guidelines recommend a maximum noise level of 52 to 55 dB(A) for call centres to prevent adverse events [19–21]. However, the French National Research and Safety Institute (INRS) has shown that call centres operators may be exposed to noise levels that exceed these guidelines [5].

The impact of noise on the health of call centres operators has been widely studied and it is even considered as one of the harmful hazards, but little attention was given to noise assessment in EMDC especially since they are not really comparable to these call centres. Indeed, the French National Authority for Health (HAS) defines medical regulation as a medical act performed over the telephone [22]. EMD and emergency physicians deal with potentially life-threatening situations. In addition, the evaluation of the patient’s condition can only be done indirectly. It is therefore a very stressful and complex intellectual activity. Stress is moreover significant because of the degree of urgency and the impact of each decision on the evaluation and management of patients. Thus, we assessed the noise level in the EMDC of the SAMU 25, before and after implementing a visual noise indicator.

## Methods

We conducted a single centre prospective observational study in the EMDC of Besancon University Hospital (France).

### Noise level measurement in the EMDC of the SAMU25

The ambient A-weighted noise level was measured using a SoundEarII® noise indicator (Dräger Medical SAS, France) approved for indoor and outdoor noise level measurement (frequency: 20 Hz to 16 kHz, scope of measurement: 40 dB(A) to 115 dB(A), deviation: +/− 3 dB(A)). This device can generate light alerts, when the sound level exceeds predetermined thresholds. We fixed this device at a central point of the EMDC in order to be visible to the whole room. The average ambient noise level was continuously measured for each minute during a 6-day period, from 00:00 to 11:59 PM, with two phases of three consecutive days. At baseline, in phase 1, the device measured noise level without visual indication. In phase 2, the indicator was visible for each one in the room and the sensor warned the staff when the noise level exceeded the pre-set thresholds: the light turned green below 65 dB(A), orange when it exceeded the first predetermined threshold (65 dB(A)) and red when noise intensity exceeded the second threshold (75 dB(A)). We chose higher thresholds than those used in the French and international guidelines. The 65 dB(A) threshold is a recommended limit to ensure proper working conditions in call centres [15]. The 75 dB(A) is the value bellow which continuous and/or repetitive exposure is unlikely to have adverse effect on the health and safety of workers [23]. The acoustic data were analysed using the software SoundLog®.

The most common epidemiological indicators of noise exposure at the workplace are “equivalent continuous sound level” (L_Aeq.T_) and percentile levels [24]. L_Aeq_ represents the averaged sound energy measured over a stated period of time T. For each phase, L_Aeq, T_ was calculated from 7:00 AM to 9:00 PM (L_Aeq, day_) and from 9:00 PM to 07:00 AM (L_Aeq, night_) according to French working time directives definitions and during the entire phase. Time spent above the INRS threshold (average background noise level at 52 dB(A)) and above the French and International organization for standardization (ISO) standards thresholds (55 dB(A)) were quantified.

Five additional conventional noise level indicators were calculated: minimum and maximum level achieved during the time of recording (L_min_ or L_max_, respectively), background noise (L_90_, exceeded sound level 90% of the interval of time of the measurement), median noise (L_50_, exceeded sound level 50% of the interval of time of the measurement) and noise of crest (L_10_, exceeded sound level 10% of the interval of time of the measurement).

### EMDC’s activity

The activity of our EMDC was estimated by the number of calls in progress during each recorded minute. We collected these data from Centaure 15®, a computerized software for file recording that is used in several EMDC in France. Based on the EMD schedules, we also considered the number of persons on duty for different periods of time (called time blocks). Each new time block began when the number of planned EMDC workers changed, and 46 different time blocks were defined for the 6 days (the time length ranged from 30 to 240 min).

### Statistical analysis

A complex structure of the data was defined as follows: the noise level was measured and analysed at each minute. The number of calls was also defined at the minute level. The number of EMDC workers was defined at the time blocks level. The duration of exposure above thresholds was defined as the proportion of time spent above the 52 and 55 dB thresholds over the course of each of these time blocks.

To analyse the potential impact of the visual noise indicator on noise level (dependent variable L_Aeq_), two-level mixed linear regression models were fitted: level 1 was defined by each minute of measurement and level 2 was defined by the time block. The analyses were adjusted on day and night periods, number of calls in progress, and number of planned EMDC workers. Interactions between number of calls and number of EMDC workers were tested.

The statistical analysis was conducted using SAS 9.4 (SAS Institute, Cary NC) and MlwiN 2.24 (Centre for Multilevel Modelling, University of Bristol).

## Results

### Ambient noise levels

**Fig. 1:**
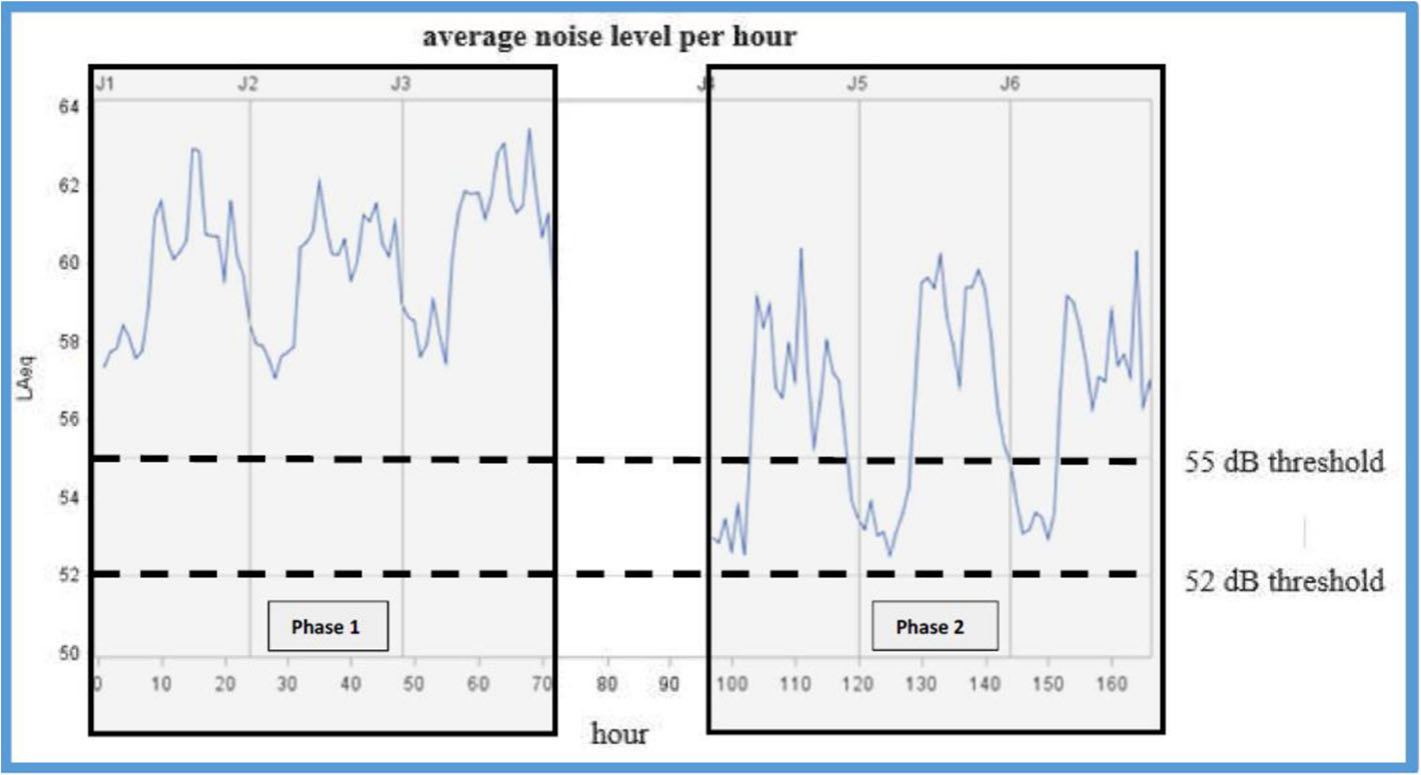
Average sound level per hour (LAeq) time evolution

Overall noise levels during the two phases are presented in Table 1 and Fig. 1. During the 3 days of phase 1, the L_Aeq_ was higher than 52 dB(A) in 97.2% of the time, compared to 66.8% during the 3 days of phase 2., L_Aeq_ was higher than 55 dB(A) in 84.9% of the time during phase 1 compared to 43.9% during phase 2.

**Table 1:** Noise levels indicators in phase 1 and phase 2 (dB(A))

Noise level (dB(A))	L_**Aeq**_	L_**max**_	L_**min**_	L_**90**_	L_**50**_	L_**10**_
**Phase 1**	60.3	76.5	48.5	54.2	58.9	62.8
**Phase 2**	56.9	71.6	47.8	48.5	54.2	60.4

L_10_: exceeded noise level for 10% of the measurement period (crest noise)

L_50_: exceeded noise level for 50% of the measurement period (median noise)

L_90_: exceeded noise level for 90% of the measurement period (background noise)

The L_10_ and L_90_ are extensively used for impulsive sound levels and Background Noise respectively

L_Aeq_ = Equivalent Sound Level. It quantifies the noise environment to a single value of sound level for any desired duration. This descriptor correlates well with the effects of noise on people

Lmax = Maximum Sound Level: a maximum level during the measurement period

Lmin = Minimum Sound Level during the measurement period

### Association between noise level and activity

Table 2 presents the L_Aeq_ according to the phase 1 or 2 (absence/presence of visual indicator), the day/night period, the number of calls and the number of EMD workers.

**Table 2:** Ambient noise level (L_Aeq_) according to intervention, night and day period, and activity

	N^a^	Mean L_Aeq_ (dB)	SD^b^
*Intervention effect*			
Phase 1	4320	58,7	3,5
Phase 2	4320	54,4	4,4
*Period*			
Day (07:00 AM – 09:00 PM)	5040	57,6	4,5
Night (09:00 PM – 07:00 AM)	3600	55,1	4,2
*Number of calls per minute:*			
0	2310	55,8	4,4
1–2	4307	56,4	4,5
≥ 3	2023	57,8	4,4
*Number of EMDC workers*			
8–9	2850	54,8	4,0
10–17	4470	56,8	4,5
18–20	1320	59,8	3,6

^a^Number of noise level measurements

^b^*SD* Standard deviation

The results of the two-level regression modelling are presented in Table 3. In the presence of the visual noise indicator (phase 2), the L_Aeq_ was significantly lower than in the absence of visual noise indicator (phase 1) (*P* < 10^–3)^. The linear regression coefficient can be interpreted as a mean difference of – 4.28 dB (Model 1). In multivariable analysis, this difference remained similar (Models 2 and 3). The L_Aeq.T_ increased significantly with the number of EMDC workers regardless of the workload (Table 4). The number of calls differed significantly between the phase 1 and the phase 2 (Table 5, *P* < 10^− 3^). The average number of calls per minute declined from 2.5 by day to 1 during night time slots in phase 1 (*p* < 10^− 3^).

**Table 3:** Multilevel models parameter estimates for ambient noise level L_Aeq_

	Model 1			Model 2			Model 3		
	β	95%CI	P	β	95%CI	P	β	95%CI	P
Fixed Part									
*Intercept*	58.93			56.83			56.8		
***Intervention effect***									
Phase 1 (without visual signal)	Ref		< 10^−3^	Ref		< 10^−3^			< 10^−3^
Phase 2 (with visual signal)	−4.28	[−5.04; − 3.52]		−4.19	[−4.63; − 3.74]		− 4.19	[− 4.63; − 3.75]	
***Period***									
Night (09:00 PM – 07:00 AM)				Ref		< 10^− 3^	Ref		< 10^− 3^
Day (07:00 AM – 09:00 PM)				1.51	[1.17; 1.85]		1.53	[1.19; 1.87]	
***Number of calls per minute***									
0				Ref		0.74			0.97
1–2				−0.03	[−0.22; 0.17]		0.03	[−0.25; 0.31]	
≥ 3				0.06	[−0.2; 0.31]		0,00	[−0.58; 0.58]	
***Number EMDC workers***									
8–9				Ref		< 10^−3^			< 10^− 3^
10–17				1.41	[0.88; 1.94]		1.36	[0.77; 1.95]	
18–20				1.72	[0.79; 2.64]		2.76	[1.6; 3.92]	
***Tnteraction between the number of calls per minute and the number of EMDC workers***							Ref		
*0 call per minute*									0.04
*1–2 calls per minute / 10–17 workers*							−0.01	[−0.41; 0.39]	
*≥ 3 calls per minute / 10–17 workers*							0.24	[−0.43; 0.90]	
*1–2 calls per minute / 18–20 workers*							−1.1	[−1.97; −0.22]	
*≥ 3 calls per minute / 18–20 workers*							−1.16	[−2.16; −0.16]	
Random Part									
*Level2: Time block*	1.58			0.39			0.38		
*Level1: Minute*	13.93			13.88			13.87		
−2*loglikelihood:	47,398.11			47,308.62			47,298.37		
Units: Time block	46			46			46		
Units: Minute	8640			8640			8640		

*CI* Confidence Interval

**Table 4:** Ambient noise levels L_Aeq_ according to both number of call and number of EMDC workers

	Number of people on duty		
Number of calls	8–9	10–17	18–20
0	56.3	59.3	63.4
1–2	56.5	59.0	61.5
≥3	57.7	59.2	61.1

The interaction between the number of calls and the numbers of EDMC workers was associated to a *P*-value lower then 10^−3^ in the two-level multivariable linear regression model

**Table 5:** Number of calls total and per minute during phase 1 and 2

Number of calls	Phase 1	Phase 2	Total
0	1101	1209	2310
1–2	1979	2328	4307
≥3	1240	783	2023
	4320	4320	8640

Chi-squared = 136.57–2 degrees of free (*P* < 10^−3^)

## Discussion

Our study highlights several issues. First of all, the visual noise indicator proved itself very useful in reducing the noise level. During phase 1, the sound levels were very high. The overall equivalent sound level (L_Aeq_) was 60.3 dB(A) during phase 1. The maximum average per minute reached up to 76.5 dB(A). In phase 1, the thresholds of 52 and 55 dB(A) respectively exceeded in 97.2 and 84.9% of the time whereas 66.8 and 43.9% of the time in phase 2. Second, the sound levels depended on the number of working people.

### Impact assessment of the noise Indicator

The visual noise indicator allowed a significant reduction of nearly 3 dB(A) in ambient noise. The sound level (L50 = 58.9 dB(A)), exceeded by 50% during phase 1, was diminished by nearly 10% in phase 2 (L50 = 54.2 dB(A)) (Table 2). Further investigation is needed to see if this user-friendly device contributes in improving the working conditions and optimal call handling and better quality of work.

### Noise level assessment

The recommended limit for background noise level is 52 dB(A) for undisturbed intellectual work [5]. The values found were greater [2, 4, 5] regardless of the considered time slot.

However, ambient noise is not the only sound perceived by the EMDs, additional noise comes from headsets too. A 20 dB(A) margin is necessary for intelligible and quality conversation. The sound level perceived directly by the EMDs could be much higher than the measured ambient noise level and may exceed the 80 dB(A) threshold requiring hearing loss preventive actions [25].

The ambient noise level within an EMDC seems comparable to that found in other call centres within the tertiary sector. It exceeded recommended limits almost all the time and sometimes even exceeded legal limits requiring preventive measures. This is worrisome. In EMDC, quick decisions, sometimes life-saving, have to be made. This requires utmost attention and focus. This is hardly compatible with a very high level of ambient noise and causes stress and exhaustion. Ambient noise can also make radio transmission less audible, leading to inaccurate assessment with possibly unfortunate repercussions.

### Factors involved in variation of sound level

The difference in sound level between the day and night time slots seemed logical and mostly due to a decrease in activity. Accordingly, the increase in activity logically leads to an increase in the sound level. In the same way, the more the people, the louder the sound. This could have several reasons. First, the mere presence of a higher number of people will obviously entail a greater noise nuisance. Finally, within EMDC, there are additional parasite noises arising particularly from operators’ interactions. There are indeed numerous exchanges between staff members ranging from simple verbal instructions without using communication headsets, to discussions mostly unrelated to work.

This partly explains the rise of the additional noise disturbances with the increase in the number of people present in the closed space of the EMDC. When a large number of personnel were present in the centre, the noise level was lower when the workload was high rather than low. The reason is that an increased workload does not allow any slackness and forces the staff into focussing on work only.

A reduction of this parasite noises could be achieved by raising awareness and changing behaviours (such as forbidding off-microphone interactions) or by bringing in adjustments on work premises (less noisy material, installation or improvement of acoustic treatment solutions, enhanced office space layout …) or workstations (dual headsets, sound level controller, daily exposure time limitation).

### Bias and limitations

This study has limitations. The use of a single sound level meter could inaccurately reflect the individual exposure to noise. Implementing different sound level meters could enhance accuracy in the measurements.

The sound level meter only gave equivalent continuous sound level for each minute. There are several fluctuating indoor and outdoor noise sources responsible for intense, punctual and significant short-term sound level increases in the EMDC: ambulances with sirens and helicopter landing and taking-off. They can exceed 120 dB(A) and be responsible of acoustics shocks. They are sources of stress but also of at least temporary hearing disorders. The most stressful and troublesome noises for the operators did not appear in our measures due to the time-averaged data processing.

Another limitation is the short duration of recording (3 days for each phase). In addition to a possible problem regarding the representativeness of the recorded period, a possible “novelty” effect could not be ruled out. The staff’s attention, considerable at the beginning of the device installation, could have diminished over time and led to a return to the previous sound level.

## Conclusion

To our knowledge, this pilot study is the first to consider the noise level in an EMDC. Such noise levels are frequently higher than the standards and sometimes close to the recommended limits, requiring preventive measures. The noise indicator was associated to a substantial reduction in ambient noise. This might have had a positive effect on staff behaviour. Further multicentric studies are needed using a more efficient noise measurement system to take into consideration many other acoustic factors and confirm our findings. This might allow the implementation of effective prevention solutions to improve operators’ well-being.

## Data Availability

The datasets generated and/or analysed during the current study are not publicly available, but are available from the corresponding author on reasonable request.

## Abbreviations

dB(A): A-weighted decibels
EMD: Emergency Medical Dispatchers in english
EMDC: Emergency Medical Dispatch Centres
HAS: Haute Autorité de Santé (French National Authority for Health)
INRS: Institut National de Recherche et Sécurité (French National Research and Safety Institute)
ISO: International Organization for Standardization
L_10_: exceeded noise level for 10% of the measurement period (crest noise)
L_50_: exceeded noise level for 50% of the measurement period (median noise)
L_90_: exceeded noise level for 90% of the measurement period (background noise)
L_Aeq_: Equivalent Sound Level
L_Aeq.T_; L_Aeq, day_: L_Aeq.T_ from 7:00 AM to 9:00 PM
L_Aeq, night_: L_Aeq.T_ from 9:00 PM to 07:00 AM
L_max_: Maximum Sound Level
L_min_: Minimum Sound Level
SAMU25: Service d’Aide Médicale Urgente de Besançon
